# Chromosome-level draft genome assembly of *Hypomesus nipponensis* reveals transposable element expansion reshaping the genome structure

**DOI:** 10.3389/fgene.2025.1502681

**Published:** 2025-04-29

**Authors:** Chenzhao Zhu, Youyi Kuang, Zhe Li, Fujiang Tang

**Affiliations:** ^1^ College of Fisheries and Life Science, Shanghai Ocean University, Shanghai, China; ^2^ Scientific Observing and Experimental Station of Fishery Resources and Environment in Heilongjiang River Basin, Ministry of Agriculture and Rural Affairs, Heilongjiang River Fisheries Research Institute of Chinese Academy of Fishery Sciences, Harbin, China

**Keywords:** *Hypomesus nipponensis*, genome, evolutionary, gene family, repeat sequence

## Abstract

*Hypomesus nipponensis* a commercially valuable fish within the Osmeriformes order, is naturally found in northeastern Asia and has been extensively introduced for commercial purposes across eastern Asia. To investigate the taxonomic status and evolutionary history of *Hypomesus nipponensis* within the Osmeridae family, we first performed a *de novo* genome assembly using PacBio HiFi reads and CLR (Continuous Long Read) reads. Subsequently, we leveraged synteny information from closely related species to further refine the assembly and construct a chromosome-level genome. The final assembly spans 507.8 Mb, with a scaffold N50 of 20 Mb, achieving chromosome-level contiguity. It comprises 164 Mb of repetitive sequences and encodes 27,876 protein-coding genes. Compared to previous assembly, the *H. nipponensis* genome is notably more contiguous and complete. Notably, it contains an unusually high proportion of tandem repeats, which likely contributed to the assembly challenges encountered in earlier efforts. We also observed the transposons of *H. nipponensis* have expanded significantly in recent times, and paralogous gene families have expanded during the same period. Our analysis estimates that *H. nipponensis*, *Osmerus eperlanus*, and *Hypomesus transpacificus* diverged from a common ancestor approximately 24.1 million years ago, with significant chromosomal segment recombination events occurring during their divergence. Additionally, we compared the genomes of *O. eperlanus* and *Hypomesus* and found that most of the genes in the Presence/Absence Variants (PAVs) of *O. eperlanus* were associated with immune response. Our efforts significantly enhance the genome’s integrity and continuity for this ecologically and commercially important fish, providing a chromosome-level genome draft that supports fundamental biological research while offering insights into the evolutionary relationships and genomic diversity within the Osmeriformes order. This advancement has profound implications for understanding the evolutionary history and adaptive strategies of *H. nipponensis*.

## Introduction

*Hypomesus nipponensis* McAllister is a small, cold-water fish species characterized by a short life cycle, high fecundity, and rapid population growth. These traits that enhance contribute to diverse environmental conditions. This species belongs to the genus *Hypomesus*, the most species-rich genus in the smelt family (Osmeridae), which currently comprises five recognized species. During the juvenile stage, most individuals migrate to the sea (sea migratory type), while others remain resident in freshwater lake (lake dwelling type) (Asami, 2004). This dual life history strategy not only facilitates colonization of divergent habitats but also promotes genetic divergence and local adaptation. Originally distributed across Japan, the Korean Peninsula and Primorsky Krai, Far East of Russia this species has been intentionally introduced to various water systems. It has subsequently become both an economically important aquaculture species and a high-quality food source for piscivorous fish (Swanson et al., 2000). Before the 1980s, *Hypomesus nipponensis* was introduced into northeastern China. In the 1980s and 1990s, it was introduced from northeastern China to a wide range of inland regions, including the highland lakes of southwest China (Xie et al., 1992). As a highly mobile species, *H. nipponensis* readily disperses between aquatic systems and is now widely distributed across northeastern Asia, including China, Japan, and the Korean Peninsula (Yin et al., 2021).

In recent years, the genome of *H. nipponensis* was assembled to investigate molecular response mechanisms to heat stress (Xuan et al., 2021). However, while the article reports a genome size of 486 Mb, only 34.4 Mb of scaffolded sequences are publicly available, indicating potential incompleteness in assembly. Incomplete genome assemblies can lead to the partial or complete omission of genes, resulting in artifacts of pseudo-gene loss that may bias downstream functional analyses (Kim et al., 2022). Integrating genomic architecture with ecological performance is therefore critical for understanding how *H. nipponensis* adapts to rapid environmental change. To date, molecular biology and genomic research on *H. nipponensis* have been limited due to the absence of a complete genome and comprehensive annotation. This lack of genomic information significantly restricts studies on the phylogeny and genetic differentiation of *H. nipponensis*. Furthermore, it hampers the exploration of the adaptation and reproductive strategies of *H. nipponensis* at the genomic level. In this study, we reassembled the *H. nipponensis* genome using PacBio HiFi sequencing and CLR data, achieving significantly improved continuity and completeness. This high-quality genome assembly enables more accurate identification and characterization of key genes, regulatory elements, and structural variations, which are essential for understanding the species’ unique biological traits and adaptive mechanisms. To better understand the evolutionary processes of *H. nipponensis*, we conducted a direct comparison with the genomes of *Hypomesus* and *O. eperlanus*. Additionally, we created chromosome collinearity maps for several species and reconstructed the ancestral chromosomes of Osmeriformes. These comprehensive analyses not only provide a robust foundation for comparative genomic studies with related species but also offer valuable insights into the ecological adaptations and evolutionary pressures shaping *H. nipponensis*’ genome.

## Results and discussion

### Chromosome-level genome assembly

**FIGURE 1:**
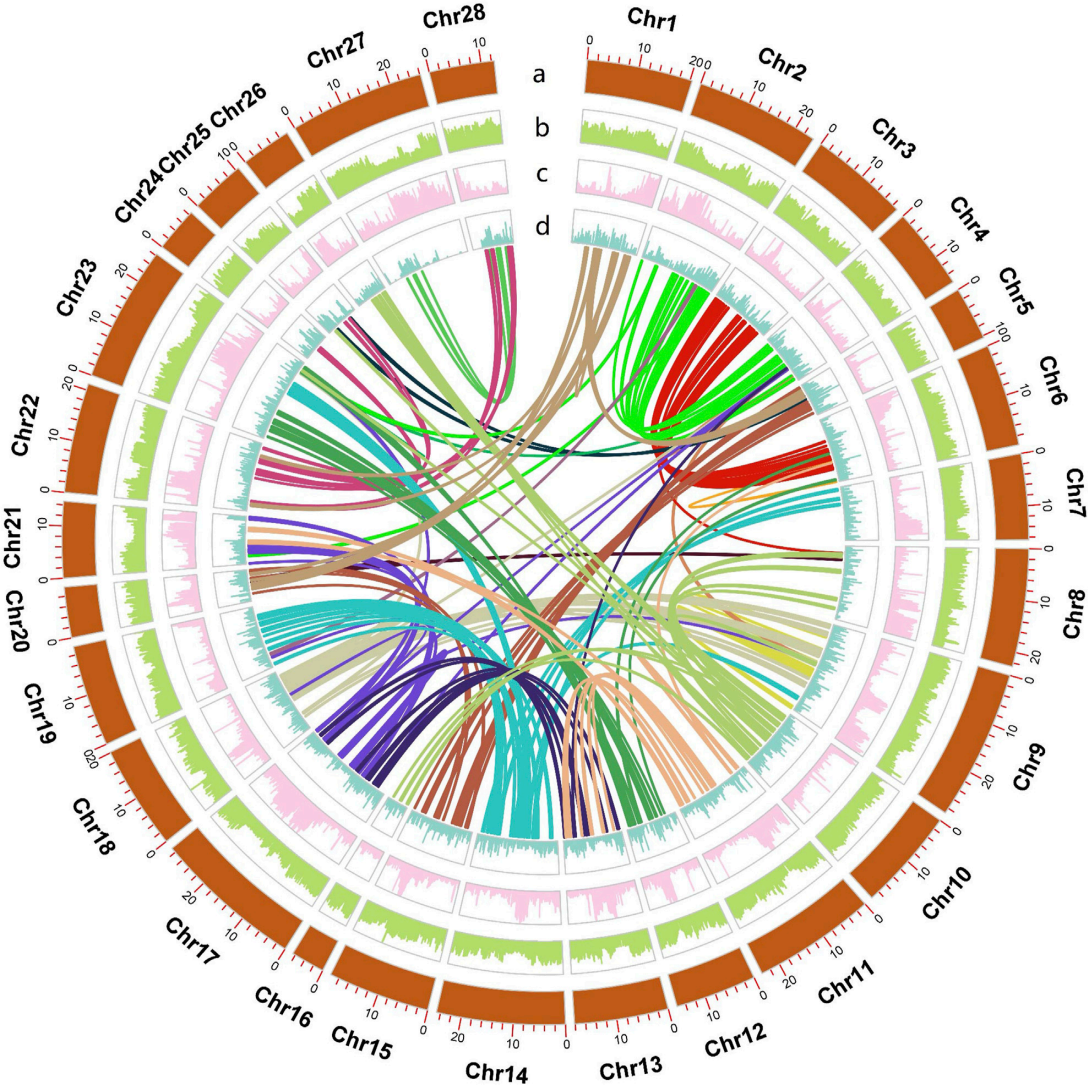
Circos plot of the *Hypomesus nipponensis* genome, with lines of different colors in the diagram represent the colinearity within the genome. The rings from inside to outside indicate (a) pseudochromosome length of the *Hypomesus nipponensis* genome (b) GC density (c) TE density, and (d) gene density; b-d were drawn in 100-kb sliding windows.

We assembled the genome using a pipeline listed in Supplementary Figure S1. First, we sequenced the genome using the PacBio platform and acquired 26.88 Gb of PacBio HiFi reads at a depth of 50×. The estimated genome size of *H. nipponensis* based on HiFi reads is approximately 463.49 Mb, with a predicted heterozygosity rate of 0.396% (Supplementary Figure S2). We generated Draft Genome v0.1 with a scaffold N50 of 0.5 Mb and Draft Genome v0.2 with a scaffold N50 of 2.3 Mb. Subsequently, we merged the two scaffolds to obtain Draft Genome v0.3, resulting in a merged scaffold N50 of 7.9 Mb. The merged sequence was polished to remove redundancy, yielding Draft Genome v0.4 with a scaffold N50 of 8.1 Mb. We then scaffolded the purged genome with CLR reads to obtain a draft genome v0.5 with a scaffold N50 of 8.1 Mb (Supplementary Table S1; Supplementary Figure S1). Finally, we constructed 28 pseudochromosomes (Figure 1). The assembly achieved a contig N50 of 8 Mb and scaffold N50 of 20 Mb, with the longest contig spanning 21 Mb and an average contig length of 1.3 Mb (Table 1). The combination of PacBio HiFi and CLR reads leverages the strengths of both technologies: HiFi provides high accuracy (∼0.1% error rate) and moderate read lengths (∼15 kb), while CLR offers longer reads, albeit with higher noise (Logsdon et al., 2020; Nurk et al., 2022; Wenger et al., 2019). However, HiFi-based assemblies often fragment at large and homogeneous repeats as well as known sequence-specific coverage dropouts. Our assembly strategy, integrating both methods, resulted in a highly contiguous and accurate genome and has been validated in the tomato genome assembly (Alonge et al., 2022).

**TABLE 1:** Statistics of the *Hypomesus nipponensis* genome assembly and corresponding gene prediction and functional annotation.

Global statistics	Genome	Gene models with evidence
Genome assembly		
Number of contigs	186	
Total contig length (pb)	532,605,080	
Estimated genome size (pb)	478,351,723	
Contig length N50 (pb)	8,193,377	
Scaffold N50 length (pb)	20,113,295	
Longest contig (pb)	21,290,931	
Average contig length (pb)	2,863,468	
GC content (%)	45.84	
N’s per 100 kbp	3.55	
BUSCO statistics (%)		
BUSCO (Actinopterygii) complete	96.7	97.2
Complete and single-copy	94.6	94.7
Complete and duplicated	2.1	2.5
Fragmented	1.1	1.1
Missing	2.2	1.7
Genome annotation		
Protein-coding gene number	27,876	
Average gene length (bp)	8,122.5	
Mean CDS length (bp)	1,541.61	
Longest CDS (bp)	36,194	
Mean protein length (aa)	513.8	
Longest protein (aa)	12,064	
Exon count per gene	8.9	
Average exon length (bp)	175.57	
Functional annotation		
Swissprot	23,764	
Gene Ontology terms	17,635	
Kegg	17,094	
TrEMBL	26,718	
Interpro	23,041	

The assembled genome exhibited high completeness, with a BUSCO (actinopterygii_odb10) score of 96.7% (including 2.3% duplicated genes) (Table 1). To further assess accuracy, we calculated the quality value (QV) of the genome, resulting in a QV of 39.4088 with an error rate of 0.0168%. The QV value of its closely related species *Hypomesus transpacificus* was 36.5925 with an error rate of 0.0219%, but the QV value of *O. eperlanus* was unknown because the original data were not uploaded. We aligned the HiFi and CLR reads to the genome, revealing that 99.92% of the genome had coverage greater than 5x. Moreover, aligning the transcriptome data to the genome resulted in a sequence alignment of 98.22% (Supplementary Table S2). These results demonstrate the high completeness and accuracy of our genome. When compared to other species of *Hypomesus*, our assembled genome exhibits superior completeness and continuity (Supplementary Table S3). The high-quality fish genome serves as a transformative key into the intricate world of aquatic life, revealing the evolutionary history, environmental adaptations, and potential applications for aquaculture (Gui et al., 2022).

### Genomic features and annotation quality

**FIGURE 2:**
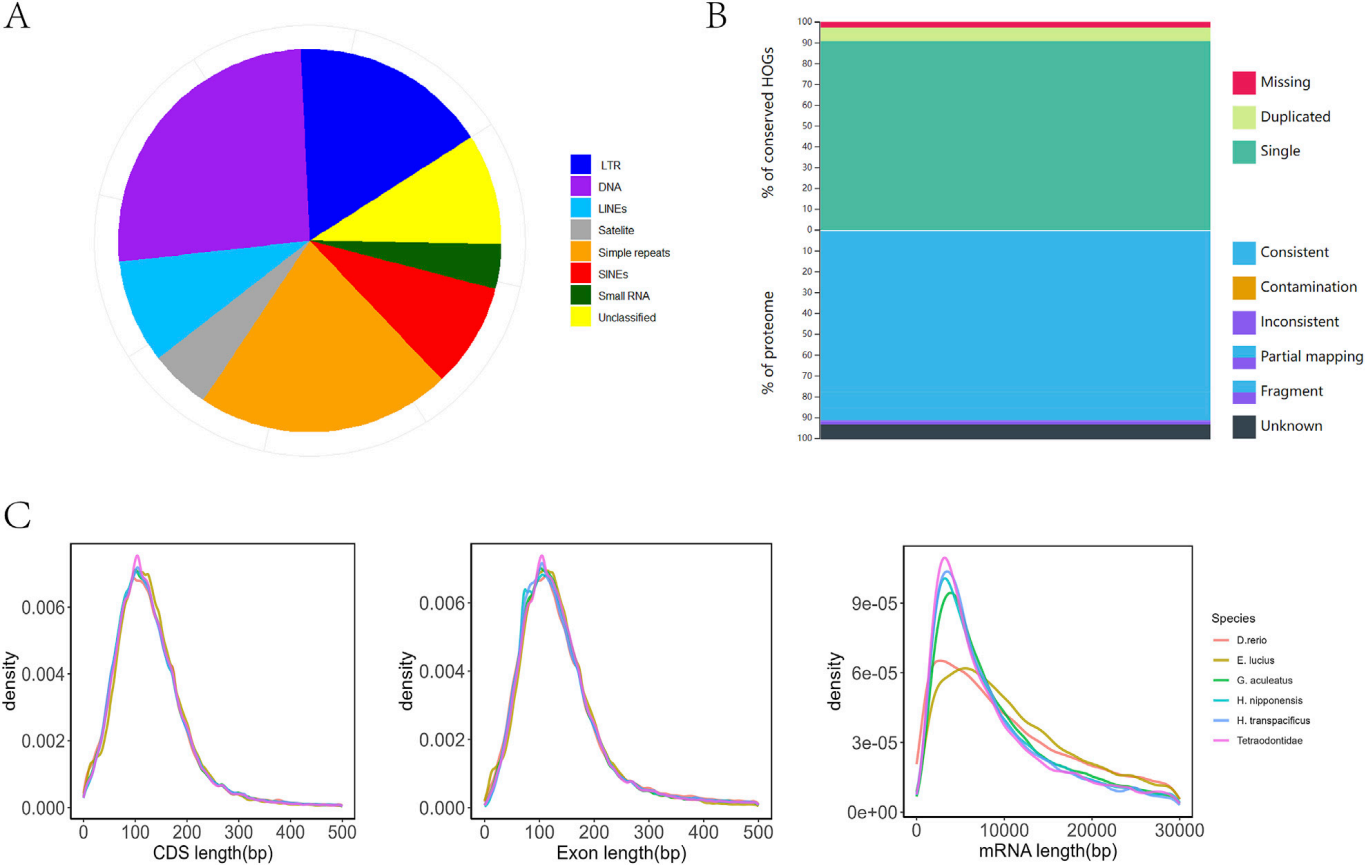
**(A)** The ratio of different repeat sequences. **(B)** The top of the stacking strip diagram represents the integrity assessment, and the lower part of the strip diagram represents the consistency assessment. **(C)** The left, middle, and right panels illustrate the distributions of mRNA length, exon length, and CDS length, respectively, in comparison with those of other representative species.

The assembled genome contains approximately 178.9 Mb of repetitive sequences, accounting for 33.59% of the total genome length. This composition included 26.02% (46.55 Mb) DNA transposons, 8.75% (15.67 Mb) long interspersed nuclear elements (LINEs), 16.69% (29.86 Mb) long terminal repeats (LTRs), and 8.75% (15.67 Mb) short interspersed nuclear elements (SINEs) (Figure 2A). The transposable elements in the genome of *H. nipponensis* are much higher than those of other fish with similar known genome sizes (*Takifugu rubripes, Gasterosteus aculeatus*), which are approximately 15% (Kasahara et al., 2007; Peichel et al., 2001; Shao et al., 2019). The high proportion of transposable elements in *H. nipponensis* suggests a dynamic evolutionary history, potentially contributing to genomic plasticity and adaptation. Similar patterns have been observed in other teleosts, where repetitive elements play a role in genome expansion and diversification (Chalopin et al., 2015). These findings highlight the importance of repetitive elements in shaping fish genomes and their functional implications.

After masking repetitive regions, we predicted 46,271 genes using *de novo* methods, 11,869–39,908 genes using homology prediction, and 22,485 genes using transcriptome prediction (Supplementary Table S4). We annotated 27,876 protein-coding genes in the assembled genome. These 27,876 genes have an average length of 8 kb and cumulatively account for 35.1% of the genome. Within the final gene set of *H. nipponensis,* 26,718 genes (95.8%) (Table 1; Supplementary Figure S3) exhibited annotated functions with at least one hit from the searched databases.

The completeness, consistency, and accuracy of the gene structure annotation were evaluated using three different strategies. First, BUSCO analysis revealed that 97.2% of the 3,640 single-copy orthologs from the actinopterygii_odb10 database were successfully identified as complete, with 94.7% classified as single-copy genes and 2.5% as duplicated genes. In contrast, 1.1% were fragmented, and 1.7% were missing in the assembly (Table 1). The OMAK assessment yielded a completeness score of 14,066 (97.34%), with 13,131 (90.87%) identified as single-copy proteins and 935 (6.47%) as duplicated proteins out of a total of 27,876 proteins (Figure 2B). Additionally, 25,310 (90.79%) proteins were consistently placed within their expected lineages. Furthermore, the length distributions of mRNA, CDS, and exons in *Danio rerio*, *Esox lucius*, *T*. *rubripes*, *G. aculeatus*, and *H. transpacificus* were found to be similar. (Figure 2C). The high BUSCO completeness score (97.2%) and low fragmentation rate (1.1%) underscore the robustness of our gene annotation, which surpasses that of the previous assembly of this species (Xuan et al., 2021). The consistency in protein placement within expected lineages (90.79%) further supports the reliability of our annotation pipeline. The similarity in mRNA, CDS, and exon length distributions across multiple species suggests conserved gene structure characteristics within teleosts, consistent with findings from other studies (Braasch et al., 2016).

### Phylogenetic placement of *Hypomesus nipponensis* and gene family analysis

**FIGURE 3:**
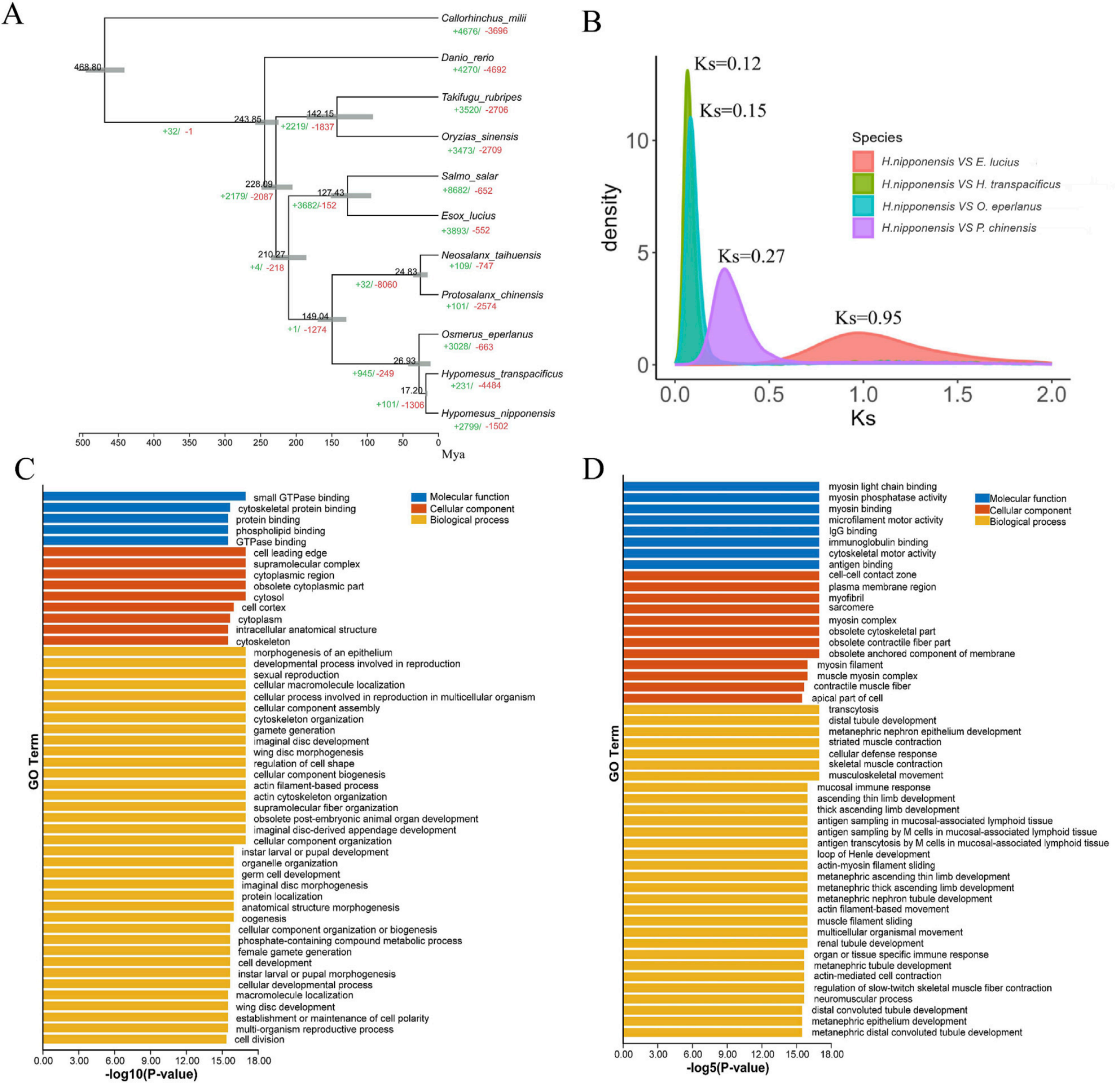
**(A)** Gene family analysis and divergence time of ten representative species, gray lines indicate confidence intervals. **(B)** The distribution of the synonymous substitution rates (Ks) of homologous genes between *Esox lucius* and *Hypomesus nipponensis*, *O. eperlanus* and *Hypomesus nipponensis*, *Hypomesus transpacificus* and *Hypomesus nipponensis*, *P. chinensis* and *Hypomesus nipponensis*. **(C)** Shows GO enrichment for expanded gene families. **(D)** shows GO enrichment for contracted.

*Hypomesus nipponensis* and ten other representative species were subjected to evolutionary and protein family analyses (Supplementary Table S6). Ultimately, we identified a total of 6,029 shared orthologous gene families, of which 586 were single-copy gene families (Supplementary Figure S4). Using these single-copy orthologous gene families, we constructed a phylogenetic tree based on the maximum likelihood method (Figure 3A). To estimate the accurate divergence time of *H. nipponensis*, we calculated the synonymous substitution rates of homoeologous genes to determine the divergence time between *Hypomesus* and *Osmerus.* The substitution rates for orthologous genes between *H. nipponensis* and various species were calculated as follows: 0.95 for *E. lucius-H. nipponensis*, 0.15 for *O. eperlanus-H. nipponensis*, 0.13 for *H. transpacificus-H. nipponensis*, and 0.27 for *P. chinensis-H. nipponensis* (Figure 3B). In addition, we calculated the synonymous substitution rates of paralogous genes in the three Osmeridae species (Supplementary Figure S5), with a peak value of Ks = 1.1. We applied the previously determined molecular clock that Ks in teleost was ∼3.51 × 10^−9^ substitutions per synonymous site per year (David et al., 2003). Therefore, the divergence time of 21.4 Mya given by synonymous substitution rates is similar to the result of the time-divergence tree, and we estimate that *H. nipponensis, O. eperlanus*, and *H. transpacificus* diverged from a common ancestor approximately 24.1 (21.4–26.9) Mya ago. Moreover, the expansion time of these three Osmeridae paralogous genes was 150 Mya, which coincides with the divergence time of Osmeridae. It is possible that the expansion of paralogous genes provided Osmeridae with a diverse gene repertoire, thereby promoting the divergence of species.

Furthermore, 231 expanded and 4,484 contracted gene families were identified in *H. nipponensis*. Notably, among the contracted gene families, those associated with sexual reproduction and gamete generation stand out (Figure 3C). In contrast, we observed significant expansions in gene families related to organ or tissue-specific immune responses, antigen sampling in mucosa-associated lymphoid tissues, and pathways associated with kidney development and skeletal muscle contraction, specifically muscle filament sliding (Figure 3D). These expansions suggest an enhancement in physical mobility and adaptability to diverse environmental challenges (Yin et al., 2021).

### Repeat expansion reshapes chromosome structure

**FIGURE 4:**
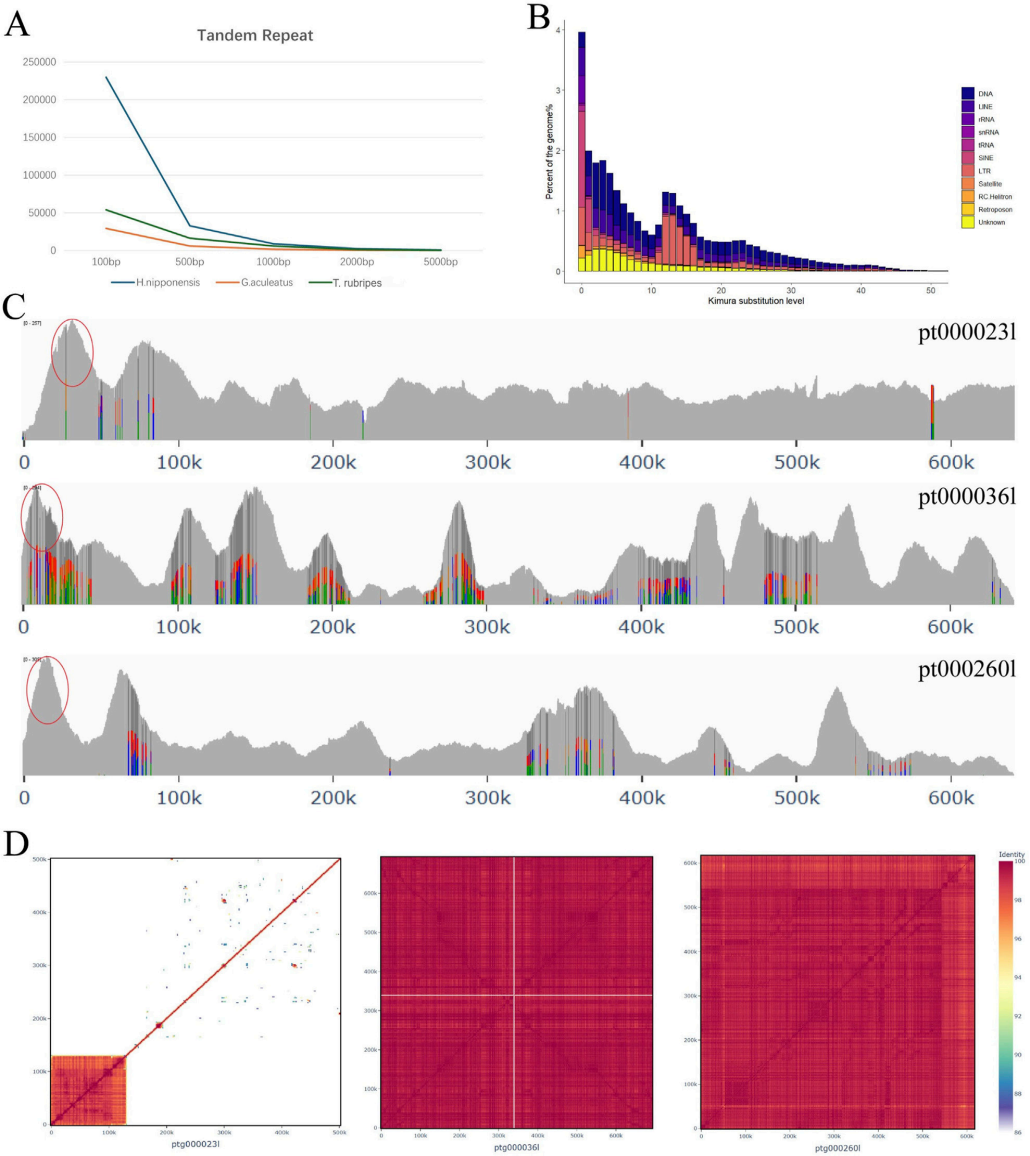
**(A)** The number of tandem repeat sequences of different lengths in *H. nippomensis*, *Gasterosteus aculeatus* and Tetraodontidae*.* **(B)** Transposable element (TE) accumulation history in the *Hypomesus nipponensis* genome, based on a Kimura distance-based copy divergence analysis of TEs, with Kimura substitution level (CpG adjusted) illustrated on the x-axis, and percentage of the genome represented by each repeat type on the y-axis. The bar color indicates the repeat type. **(C)** HiFi reads the coverage depth of three scattered sequences. **(D)** Heatmap of average nucleotide identities between pairwise combinations of genomic intervals from three sequences.

During the assembly of *H. nipponensis* genome, we observed relatively lower genome continuity. To investigate this, we compared the repeats of *H. nipponensis* with those of the *G. aculeatus* and *T*. *rubripes*. Our analysis revealed that the tandem repeat sequences constitute 3.26% of the *G. aculeatus* genome, 5.83% of the *T*. *rubripes* genome, and a significantly higher 14.61% of the *H. nipponensis* genome (Figure 4A; Supplementary Table S5). In order to explore the reasons for the increase in repeat sequences in *H. nipponensis*, we employed an analysis based on Kimura distance (Kimura, 1980), which revealed two major expansion events in the repetitive sequences of the genome (Figure 4B). The more recent peak is caused by a significant expansion of all transposons (Supplementary Figure S6), and the paralogous gene families of *H. nipponensis* were observed to have expanded recently (Supplementary Figure S5). The most recently expanded genes were identified, and two paralogues with more pronounced amplification were extracted (Supplementary Figure S7). The contigs containing these duplicated fragments were subsequently aligned, revealing aligned bases of 7.82% and 6.34%, respectively. Furthermore, the read depth of both HiFi and CLR data was consistent with genome-wide coverage, excluding alternative haplotypes. Assembly-induced duplication artifacts were also ruled out. The repetitive sequences in these recently amplified fragments were then extracted, and a Kimura distance map was constructed for them (Supplementary Figure S8). It was found that the recent amplification of LINEs was very obvious, which was consistent with the amplification curve of the paralogous gene family in the genome, so the amplification of the repetitive sequences was likely to have led to the expansion of the gene family. Gene Ontology (GO) enrichment analysis of these genes indicated that the majority are associated with chromatin DNA binding functions (Supplementary Figure S9). The DNA transposons and LINEs peaks are similar and show enrichment around 30 Mya, which may be attributed to the differentiation of the Osmeridae family. The farther peak is caused by the expansion of LTRs, which occurred before 140 Mya (Supplementary Figure S6). This implies that *H. nipponensis* genome has experienced dynamic evolutionary changes due to the proliferation of diverse repetitive elements. The multiplication of these transposable elements likely contributed significantly to shaping the genome’s current structure.

Furthermore, we extracted some scattered contigs from the assembly results of HiFi reads, constructed a heat map of nucleotide similarity between pairwise combinations of genomic intervals (Figure 4C), and found that the proportion of repeated sequences at one end was very high. Examining these contigs, we found that the depth at one end is very high (Figure 4D), which may be the reason for the poor continuity of the genome. We then explored these duplications and found that they were all caused by SINE/5S amplification, likely associated with the most recent SINEs expansion (Supplementary Figure S6).

### Chromosomal structure and evolutionary patterns in osmeriformes

**FIGURE 5:**
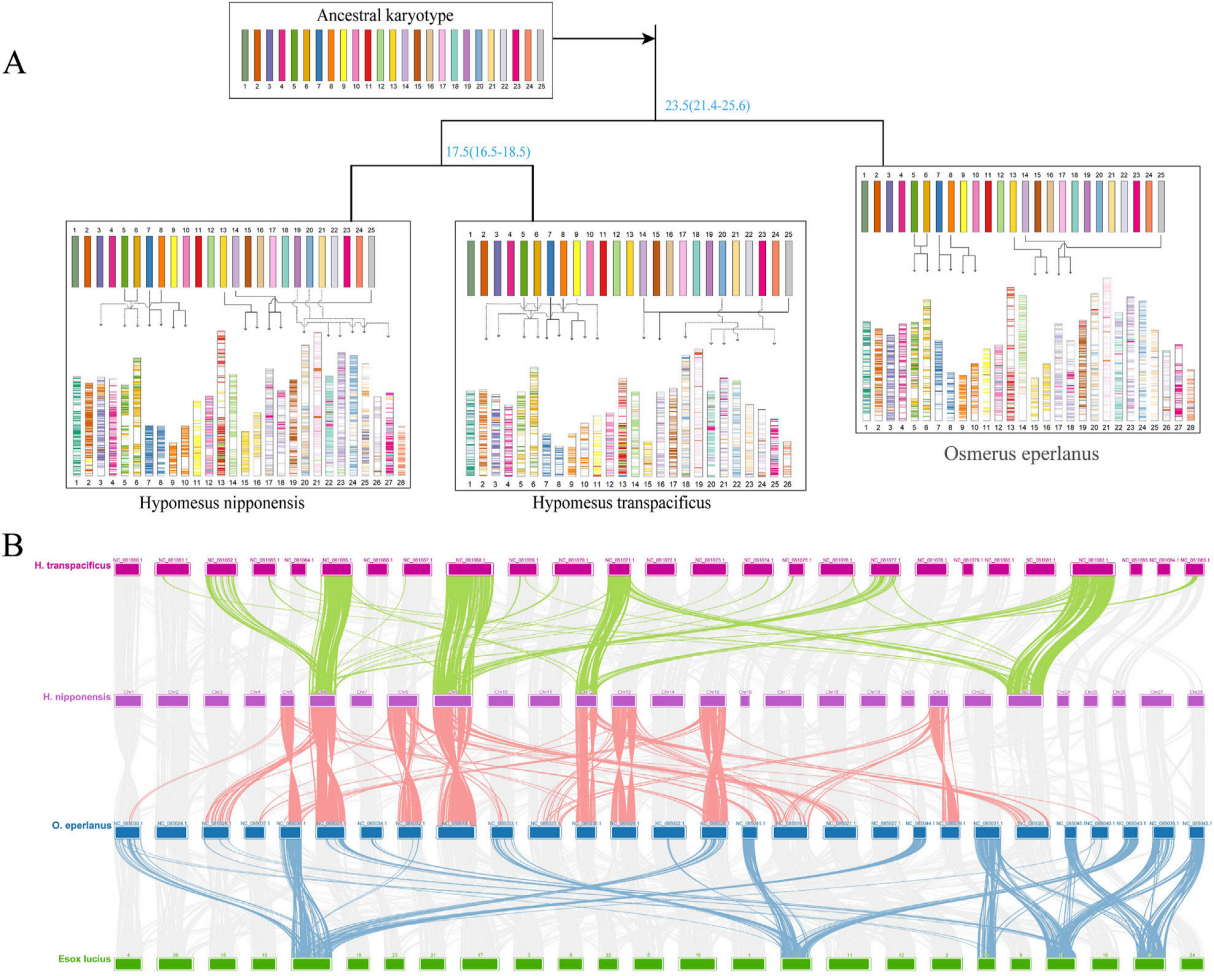
**(A)** Ancestral karyotype and chromosomal evolution of various Salmoninae species. **(B)** Collinearity analysis of *Hypomesus nipponensis*, *Esox lucius*, and *O. eperlanus* genomes.

Reconstruction of the ancestral Osmeriformes karyotype identified 25 ancestral chromosomes (Figure 5A), which aligns with prior estimates that the ancestral chromosomes of bony fish were 24 or 25 (Muffato et al., 2023; Nakatani et al., 2007). During the differentiation into *O. eperlanus*, chromosomes 7, 8, 13, and 14 experienced structural breakage; chromosomes 25 and 14 fused, resulting in the 28 chromosomes characteristic of Osmeridae. Additionally, chromosomes 5 and 6 experienced extensive recombination, as did chromosomes 23 and 14. In the lineage leading to *H. nipponensis*, beyond the aforementioned changes, chromosomes 8 and 3 recombined, and chromosome 23 further recombined with chromosome 18. *Hypomesus transpacificus* and *H. nipponensis* show similar recombination, but the reason why it has only 26 chromosomes may be due to its failure to assemble all 28 chromosomes, two of which are indistinguishable (Kitada et al., 1981). These chromosomal rearrangements—including breakages, fusions, and recombination events—appear to be key drivers in the speciation within Osmeriformes. The conserved ancestral karyotype of 25 chromosomes provides a stable basis, while lineage-specific structural modifications have likely facilitated ecological diversification and reproductive isolation. These mechanisms are consistent with observations in other teleosts, where chromosomal evolution has been linked to speciation processes (Kirkpatrick, 2010; Parey et al., 2022).

A collinearity map of *E. lucius*, *O. eperlanus*, *H. nipponensis,* and *H. transpacificus* revealed distinct chromosomal recombination patterns, with darker regions indicating higher recombination activity (Figure 5B). Notably, chromosomes 6, 8, 9, 12, 13, 15, and 21 of *H. nipponensis* and the corresponding chromosomes of *O. eperlanus* have all undergone recombination events. Furthermore, genomic comparisons indicate that most chromosomes of *H. nipponensis* and *H. transpacificus* have undergone recombination in the past 20 million years (Supplementary Figure S10). Among them, chromosomes 21, 20, and 12 exhibit both inversions and translocations, and chromosome 23 has undergone multiple inversions and translocations. In addition, the density of repetitive sequences at both ends of the inversion fragment on chromosome 12 of *H. nipponensis* is very high, as is that on chromosome 23 (Supplementary Figure S11), suggesting that these inversion events are likely mediated by repetitive sequences. Chromosomal recombination is a major driver of genomic evolution, often contributing to species diversification and adaptation. The observed chromosomal recombination events, including inversions and translocations, are likely more than mere structural variations; they may play a crucial role in the adaptive evolution of *H. nipponensis*. Enhanced recombination activity could generate novel gene combinations, thereby accelerating adaptive responses to environmental challenges such as rising water temperatures and habitat variability. Indeed, previous studies have linked chromosomal rearrangements to ecological diversification and the evolution of temperature tolerance in teleosts (Donelson et al., 2012; Wellenreuther and Bernatchez, 2018). In the case of *H. nipponensis*, the dynamic genomic architecture—evidenced by high recombination and the association with repetitive elements—may underlie its ability to thrive in diverse and changing environments. Furthermore, the correlation between regions of high repetitive sequence density and inversion breakpoints supports the hypothesis that transposable elements contribute to chromosomal instability, which in turn may facilitate rapid adaptation (Chalopin et al., 2015; Shao et al., 2019). These chromosomal modifications not only promote species diversification but also potentially enhance the capacity of *H. nipponensis* to adjust to environmental stressors, such as increasing temperatures and fluctuating ecological conditions.

We identified 42,174 segments in *Hypomesus*, with a total length of 96 Mb, that are absent in *O. eperlanus*, and 48,296 segments in *O. eperlanus*, with a total length of 108 Mb, that are absent in *Hypomesus* (Supplementary Figure S12). Gene enrichment in the *O. eperlanus* PAVs regions revealed that they were significantly enriched in the immunological memory process pathway (GO:0090713) and the positive regulation of the protein secretion pathway (GO:0050714) (Supplementary Figure S13). The comparative genomic analysis highlights the genomic diversity within Osmeriformes and provides insights into the adaptive evolution of these species. The enrichment of immune-related pathways in *O. eperlanus* PAVs suggests that its migratory lifestyle, which likely exposes the species to diverse pathogens and environmental stressors, has driven the expansion of immune-related genes. This finding is consistent with studies in migratory fish, where immune system adaptations are critical for survival in variable environments (Hopkins II and Warren, 2005). The identification of PAVs also underscores the importance of structural variations in shaping species-specific traits and ecological adaptations.

## Materials and methods

### Sample collection, library construction, and genome sequencing

*Hypomesus nipponensis* muscle sample was collected from the Yalu River in Dandong (N 40.51, E 124.97), China, for whole genome sequencing. High-quality genomic DNA was extracted using optimized Cetyl Trimethyl Ammonium Bromide protocol. A 15 kb SMRTbell library was constructed using the SMRTbell Express Template Prep Kit 3.0 (Pacific Biosciences, CA, United States), following standard protocols for DNA shearing, damage repair, end repair, hairpin adapter ligation, size selection, and purification. The library was sequenced on the PacBio Revio platform (25 M SMRT Cell) in Circular Consensus Sequencing (CCS) mode to generate high-fidelity (HiFi) reads with greater than 99.9% accuracy.

### Genome size estimation

KmerGenie version 1.7051 (Chikhi and Medvedev, 2014) was used to perform *k*-mer counting and determine the optimal *k*-mer size for downstream analysis. The optimal *k*-mer size was estimated to be 119 bp, as it provided a balance between read coverage and specificity for accurate *k*-mer profiling. The *k*-mer frequency output from KmerGenie was then used as input to GenomeScope (Vurture et al., 2017) for genome size estimation.

### Genome assembly

Before assembly, we conducted a quality control on the PacBio HiFi reads. Reads with a median quality below Q20 were filtered out, and residual adapter sequences were trimmed. Only high-quality reads that passed these filters were used for the *de novo* genome assembly. This ensured the accuracy and reliability of the final assembly.

First, we used hifiasm v0.19.8 (Cheng et al., 2022) to assemble the HiFi reads, which are high-fidelity long reads generated through CCS. To further improve assembly continuity, we integrated PacBio CLR data (PRJNA672783 from NCBI) followed by additional assembly using NextDenovo v2.5.2 (Hu et al., 2023). We integrated the two assemblies using QuickMerge v0.3 (Chakraborty et al., 2016) with the hifiasm assembly as the reference. The merged sequence was then polished with NextPolish v1.4.1 (Hu et al., 2020). Following this, we removed redundancy using Purge-Dups v1.2.6 (Guan et al., 2020) and minimap2 v2.26 (Li, 2021) and subsequently employed masurca v4.1.0 (Zimin et al., 2013) to construct scaffolds. Finally, we constructed pseudochromosomes by anchoring scaffolds to the reference genomes of *H. transpacificus* (GCF_021917145.1) and *O. eperlanus* (GCF_963692335.1) using Ragtag v2.1 (Alonge et al., 2019). We employed TGS-GapCloser v1.2.1 (Xu et al., 2020) to close assembly gaps using HiFi and CLR data (Supplementary Figure S1). To assess genome completeness, we used BUSCO v5.4.6 (Simão et al., 2015) with the actinopterygii_odb10 lineage database (https://busco-data.ezlab.org/v5/data/lineages/actinopterygii_odb10.2024-01-08.tar.gz) as a reference.

### Repeat identification

We predicted repeat elements using both *de novo* and homology-based annotations. RepeatModeler v2.0.6 (Flynn et al., 2020) and EDTA v2.2.2 (Ou et al., 2019) were employed to perform *de novo* repeat prediction and construct a custom repeat library. Then, the two libraries were combined and used to annotate the assembled genome with RepeatMasker (Chen, 2004). For the homology-based prediction, the Repbase (Jurka et al., 2005) and Dfam (Storer et al., 2021) libraries were used with RepeatMasker to identify known repeat elements. Finally, data from both methods were integrated to produce a nonredundant repeat element set.

We calculated the Kimura substitution levels between repeat consensus sequences and their genomic copies using the calcDivergenceFromAlign.pl script, which is included in the RepeatMasker utility bundle. We generated repeat landscape plots with the R script Kimura_Distance_plot.R, leveraging the divsum output from calcDivergenceFromAlign.pl.

### Gene annotation

To predict protein-coding genes, we employed a combination of homology-based, *de novo*, and transcriptome-based prediction methods. Protein sequences from nine representative teleost species—*D. rerio* (zebrafish)*, Tetraodon nigroviridis* (pufferfish)*, G. aculeatus* (stickleback)*, Oryzias latipes* (medaka)*, Salmo salar* (salmon)*, H. transpacificus* (delta smelt)*, T. rubripes* (fugu)*, Oreochromis niloticus* (tilapia)*, and E. lucius* (northern pike)—were retrieved from Ensembl (Flicek et al., 2014), Gene structures were subsequently inferred using miniprot v0.12 (Li, 2023). For *de novo* gene prediction, Augustus (Stanke et al., 2008) and BRAKER3 v3.0.8 (Gabriel et al., 2024) were used on the repeat-masked *H. nipponensis* genome. RNA-seq data (PRJNA672783) from NCBI (Xuan et al., 2021) were aligned to the *H*. *nipponensis* genome using Hisat2 v2.2.1 (D. Kim et al., 2019). Transcript assemblies and transcriptome-based prediction were using TransDecoder v5.7.1 (Haas et al., 2008), StringTie v2.2.1 (Shumate et al., 2022), and PASA v2.5.3. Finally, the gene models obtained from homology-based, *de novo*, and transcriptome-based predictions were integrated using EVidenceModeler v2.1.0 (Haas et al., 2008) to construct a unified and high-confidence gene set (Supplementary Figure S1).

For functional annotation, BLASTp (Altschul et al., 1990) was used to align the predicted protein against five public databases, including SwissProt (Boeckmann et al., 2003), TrEMBL (Boeckmann et al., 2003), KEGG (Kanehisa and Goto, 2000), GO (Consortium, 2004) and InterPro (Paysan-Lafosse et al., 2023).

The completeness, consistency, and accuracy of the gene structure annotation were evaluated using three different strategies. First, BUSCO analysis was performed to assess the completeness of single-copy orthologs using the actinopterygii_odb10 database. Next, OMAK (Nevers et al., 2024) was applied to evaluate genome integrity and consistency by comparing the annotated protein sequences with those from the ancestors of Teleostei. Additionally, the length distributions of mRNA, CDS, and exons were compared among *D. rerio*, *E*. *lucius*, *T*. *rubripes*, *G. aculeatus*, and *H. transpacificus* to assess gene structural similarity and conservation.

### Orthology and phylogenomics

To investigate evolutionary relationships, *H*. *nipponensis* and ten other fish species—*D*. *rerio, S*. *salar, E. lucius, T*. *rubripes, C*. *milii, O*. *sinensis, H. transpacificus, O. eperlanus, P*. *chinensis, N*. *taihuensis*—were selected for orthology analysis. To reduce redundancy, only the longest isoform per gene was retained in each species’ protein set. Orthology inference was performed using Orthofinder v2.5.4 (Emms and Kelly, 2019), which identified orthologs, paralogs, and co-orthologs across species. For phylogenetic tree construction, we first extracted 586 single-copy orthologs, aligned their corresponding CDS sequences individually using MUSCLE v5.1 (Edgar, 2022), and then concatenated them into a supergene matrix. Iqtree v2.3.3 (Nguyen et al., 2015) was applied to construct a phylogenetic tree with the maximum-likelihood method, with 100 bootstrap replicates. Species divergence times were estimated with MCMCTree (Alvarez-Carretero et al., 2022), a component of PAML (Yang, 2007), employing the parameters ‘RootAge ≤500, model = F81, alpha = 1, clock = 3′and calibration points for *C*. *milii* and *D*. *rerio* (440–495 Mya) (Betancur et al., 2013); *D*. *rerio* and *S*. *salar* (180Mya-251.5Mya) (Meynard et al., 2012)*; S*. *salar* and *H*. *transpacificus* (176Mya-264Mya) (Rabosky et al., 2018). To visualize the consistency between the genomes of *H. nipponensis* and its closely related species, *O. eperlanus* and *E. lucius*, the 28 *H. nipponensis* chromosomes were aligned with *O. eperlanus* and *E. lucius* chromosomes by MCScanX (Wang et al., 2012).

### Expansion and contraction of gene families

We assessed gene family expansion and contraction in *H. nipponensis* by comparing cluster size differences with ten other fish species using CAFE5 (Mendes et al., 2021). We applied a stochastic birth and death model to investigate changes in gene family size along each lineage of the phylogenetic tree. A gamma model was used to estimate the probability of transitions in gene family size between parent and child nodes. We calculated P-values for each lineage using conditional likelihoods as test statistics, with a P-value less than 0.05 indicating significant gene family expansion or contraction. We annotated the protein sequences of *H. nipponensis* with EggNOG mapper (Cantalapiedra et al., 2021) as background genes, extracted all significantly expanded or contracted gene families, performed GO enrichment analysis, and generated plots using TBtools (Chen et al., 2023).

### Reconstruction of ancestral karyotype

A total of four species—*E. lucius, O. eperlanus, H. nipponensis,* and *H. transpacificus*—were selected for the reconstruction of the ancestral karyotype. *Esox lucius* was adopted as a reference genome and BLAST was used for pairwise interspecies comparisons and reciprocal best hits to obtain a set of genes homologous between species. The corrected posterior binomial test (q-value <0.05, homologous genes count ≥20) was applied to detect chromosomes that were homologous among species. The default parameters of MCScanX were used for the identification of inter-chromosomal colinear blocks. Finally, ANGeS v1.01 (Wang et al., 2012) was used to construct the ancestral karyotype. Interspecies collinearity was displayed using Circos v0.69 (Northcutt, 2009).

### Identification of PAVs

We initially constructed the genomes of *H. nipponensis* and *H. transpacificus* using ppsPCP (Tahir Ul Qamar et al., 2019). Next, we identified putative PAVs by aligning the *Hypomesus* genomes with *O. eperlanus* using Mummer v4.0.1 and extracting unaligned regions from the “show-diff” command. These sequences were then filtered by discarding those overlapping with gap regions in the respective genome. To identify putatively unique presence regions, the remaining sequences were filtered by aligning them with the other genome using BLASTN (E-value ≤ 1e-5). Sequences exhibiting high similarity (≥95%) and coverage (≥90%) were removed from the dataset.

## Conclusion and limitations

In this study, we report an improved chromosome-level draft genome of *H*. *nipponensis*, totaling 507.8 Mb with a scaffold N50 of 20 Mb, of which 96.6% of the assembly was anchored to 28 pseudochromosomes. Our analysis revealed substantial expansions in gene families involved in tissue-specific immune responses, antigen sampling in mucosa-associated lymphoid tissues, kidney morphogenesis, and skeletal muscle function—particularly muscle filament sliding—thereby highlighting potential genomic bases for physiological adaptation. Notably, the recent expansion of LINEs appears to be closely associated with gene family proliferation, suggesting that transposable elements may play a central role in genome remodeling. Furthermore, ancestral karyotype reconstruction of Osmeriformes inferred 25 ancestral chromosomes and subsequent synteny analyses revealed that lineage-specific chromosomal rearrangements (including inversions and translocations) were likely facilitated by the accumulation of repetitive sequences. These findings highlight the critical role of repeat-driven structural variation in shaping the genome architecture of *H. nipponensis*, contributing to both its evolutionary divergence and ecological adaptation.

However, despite the valuable insights gained, several methodological and interpretative limitations should be acknowledged. Most notably, the chromosome-level assembly was not supported by Hi-C chromatin conformation data but was instead constructed using a synteny-based anchoring strategy with closely related species. Although this approach facilitates the inference of large-scale chromosomal architecture, it lacks the resolution to capture long-range chromatin interactions and accurately determine scaffold orientation. This limitation may introduce potential misassemblies, particularly in regions rich in repetitive elements or structural complexity, thereby affecting the accuracy of inferred chromosomal recombination, inversion, and translocation events. In summary, while this study establishes a valuable genomic resource for *H. nipponensis* and advances our understanding of its evolutionary dynamics, the absence of chromatin conformation data (Hi-C) and experimental validation introduces uncertainty regarding both genome assembly accuracy and functional interpretation. Future studies incorporating Hi-C sequencing, long-read haplotype phasing, and functional genomic analyses will be essential to validate and refine these findings, ultimately advancing our understanding of genome evolution and adaptive diversification in Osmeriformes.

## Data Availability

The data presented in this study are deposited in the CNCB Sequence Archive of the China National Center for Bioinformation, under the BioProject accession number PRJCA024905. The chromosome-level genome assembly and annotation data are available in the Zenodo repository under accession number GWHETSC00000000.2, accessible at https://doi.org/10.5281/zenodo.14868385. All other data generated or analyzed during this study are included in the manuscript and its Supplementary Materials.
