# Supplementary material for: Chromosome-level draft genome assembly of Hypomesus nipponensis reveals transposable element expansion reshaping the genome structure
Source: Front Genet. 2025 Apr 29;16:1502681. doi: 10.3389/fgene.2025.1502681 (PMC12083275; doi:10.3389/fgene.2025.1502681)
Supplement: Supplementary file 1 [file Table1.docx]

| **Table 1**: Statistics of the *H. nipponensis* genome assembly and corresponding gene prediction and functional annotation | | |
| --- | --- | --- |
| Global statistics | Genome | Gene models with evidence |
| Genome assembly  Number of contigs  Total contig length(pb)  Estimated genome size(pb)  Contig length N50(pb)  Scaffold N50 length (pb)  Longest contig (pb)  Average contig length(pb)  GC content(%)  N's per 100 kbp  BUSCO statistics(%)  BUSCO (Actinopterygii) complete  Complete and single-copy  Complete and duplicated  Fragmented  Missing | 186  532,605,080  478,351,723  8,193,377  20,113,295  21,290,931  2,863,468  45.84  3.55  96.7  94.6  2.1  1.1  2.2 | 97.2  94.7  2.5  1.1  1.7 |
| Genome annotation |  |  |
| Protein-coding gene number | 27876 |  |
| Average gene length (bp)  Mean CDS length (bp)  Longest CDS (bp) | 8,122.5  1,541.61  36194 |  |
| Mean protein length (aa)  Longest protein (aa)  Exon count per gene  Average exon length (bp)  Functional annotation  Swissprot  Gene Ontology terms  Kegg  TrEMBL  Interpro | 513.8  12064  8.9  175.57  23764  17635  17094  26718  23041 |  |
