# Supplementary material for: Chromosome-level draft genome assembly of Hypomesus nipponensis reveals transposable element expansion reshaping the genome structure
Source: Front Genet. 2025 Apr 29;16:1502681. doi: 10.3389/fgene.2025.1502681 (PMC12083275; doi:10.3389/fgene.2025.1502681)
Supplement: Supplementary file 2 [file DataSheet1.docx]

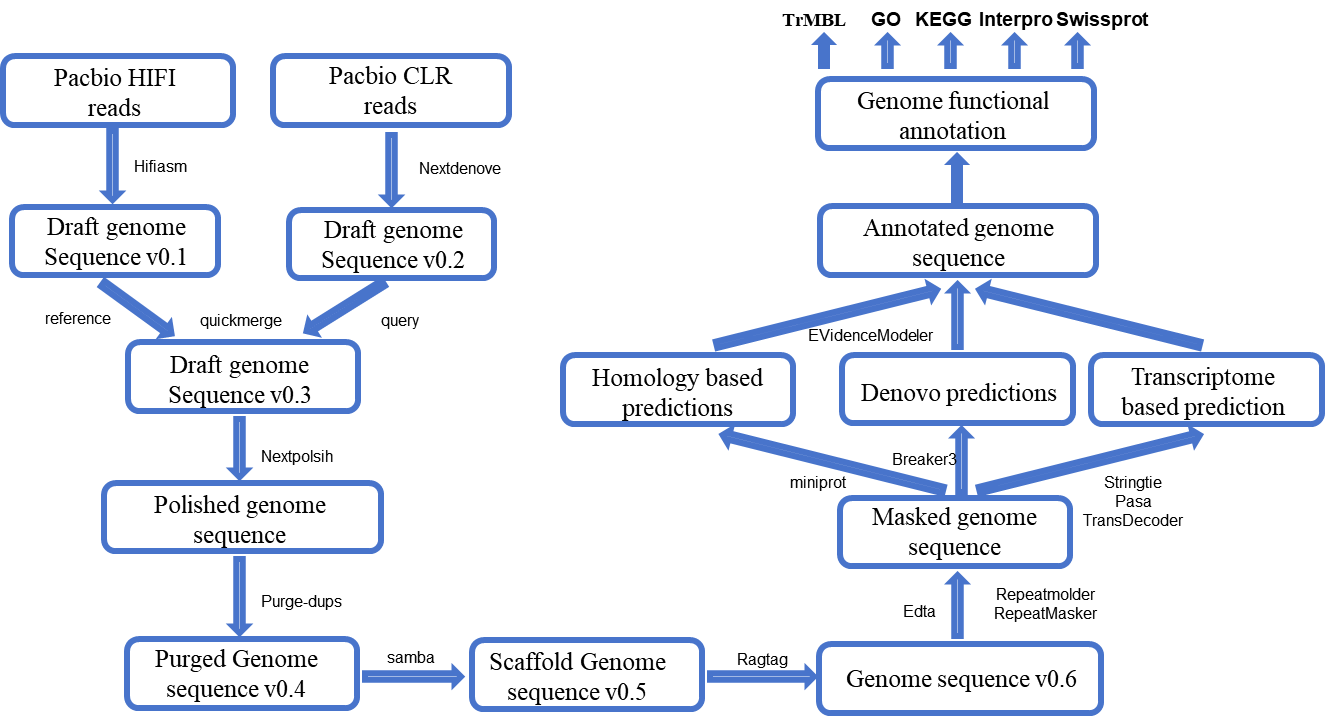
**Supplementary Figure S1.** *Hypomesus nipponensis* Genome Assembly Workflow Diagram

**
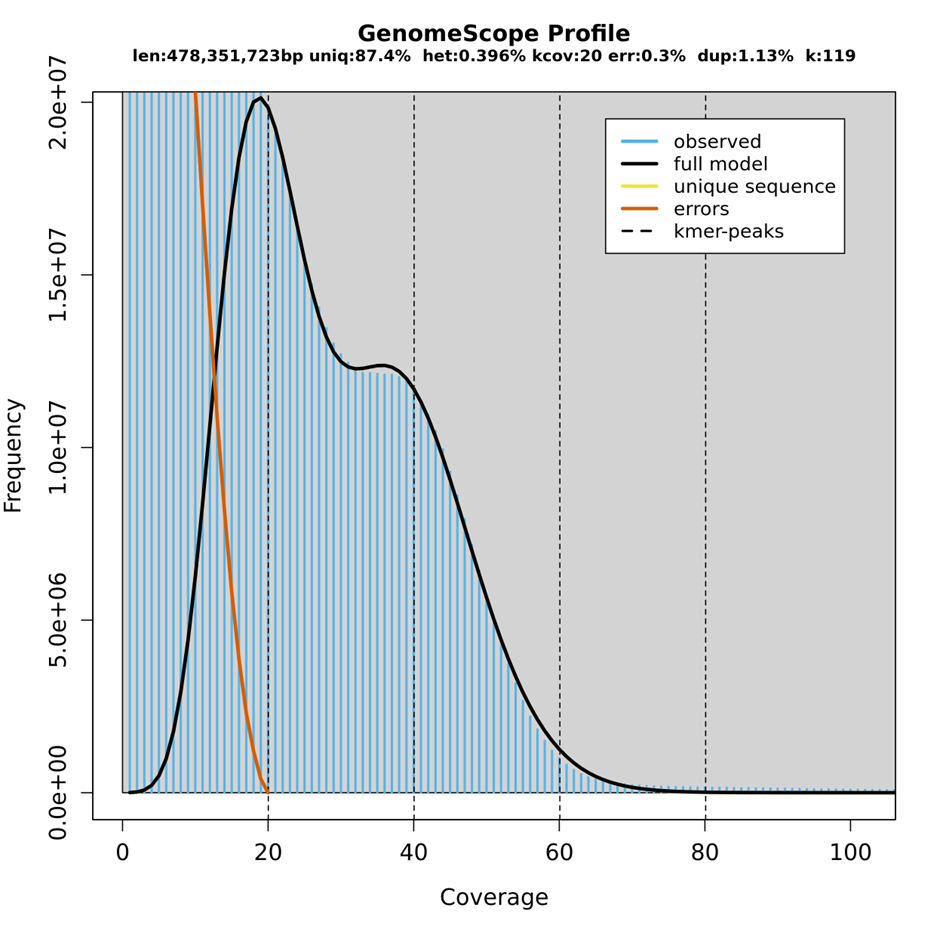
Supplementary Figure S2.** K-mer analysis of *H. nipponensis* genome.


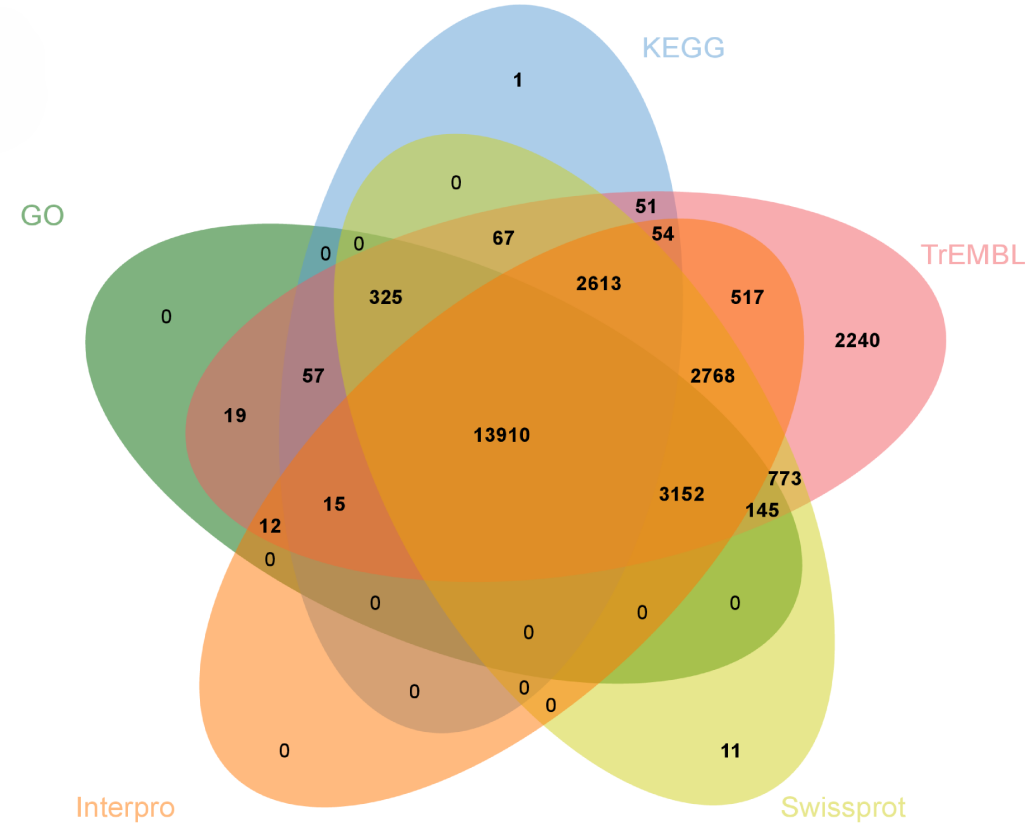
**Supplementary Figure S3.** The Venn diagram shows the shared and unique annotations among InterPro, KEGG, KOG, NR and SwissProt

**
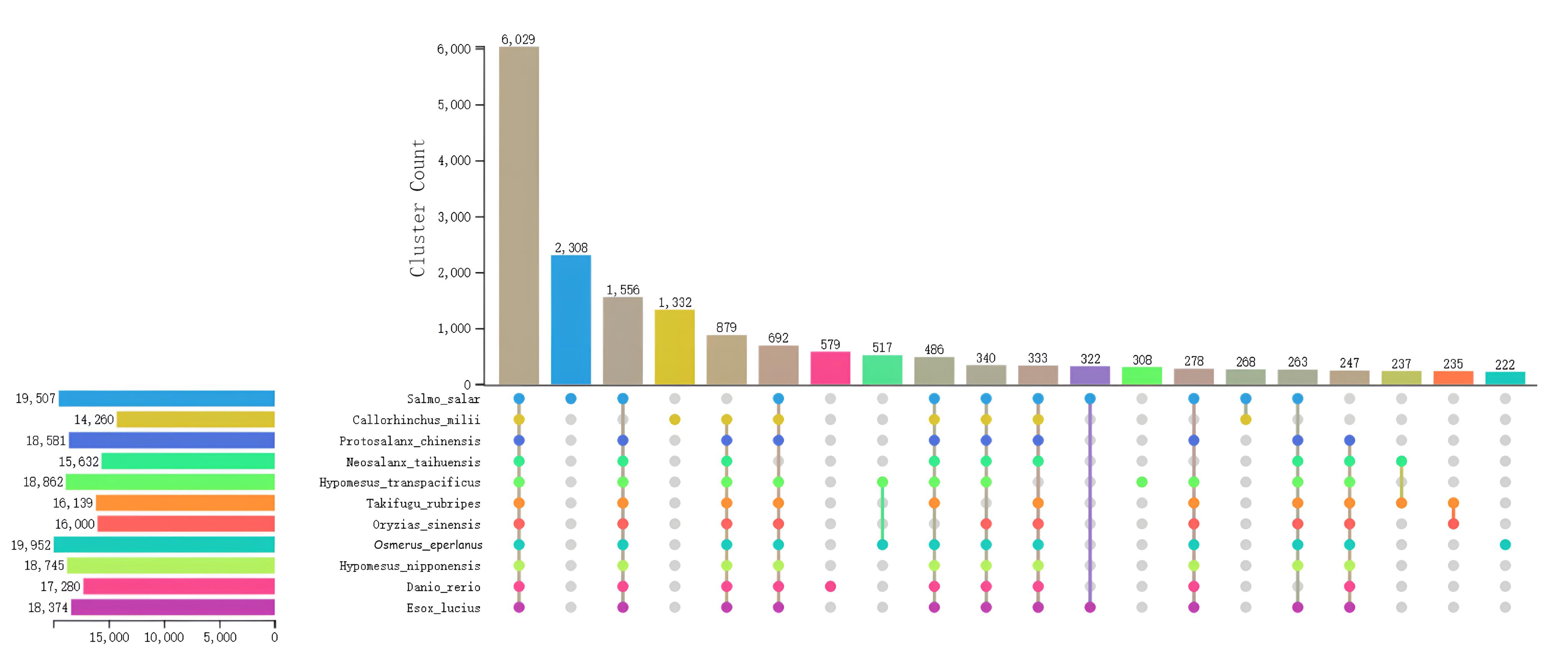
**

**Supplementary Figure S4.** Distribution of gene families in eleven fish species

**
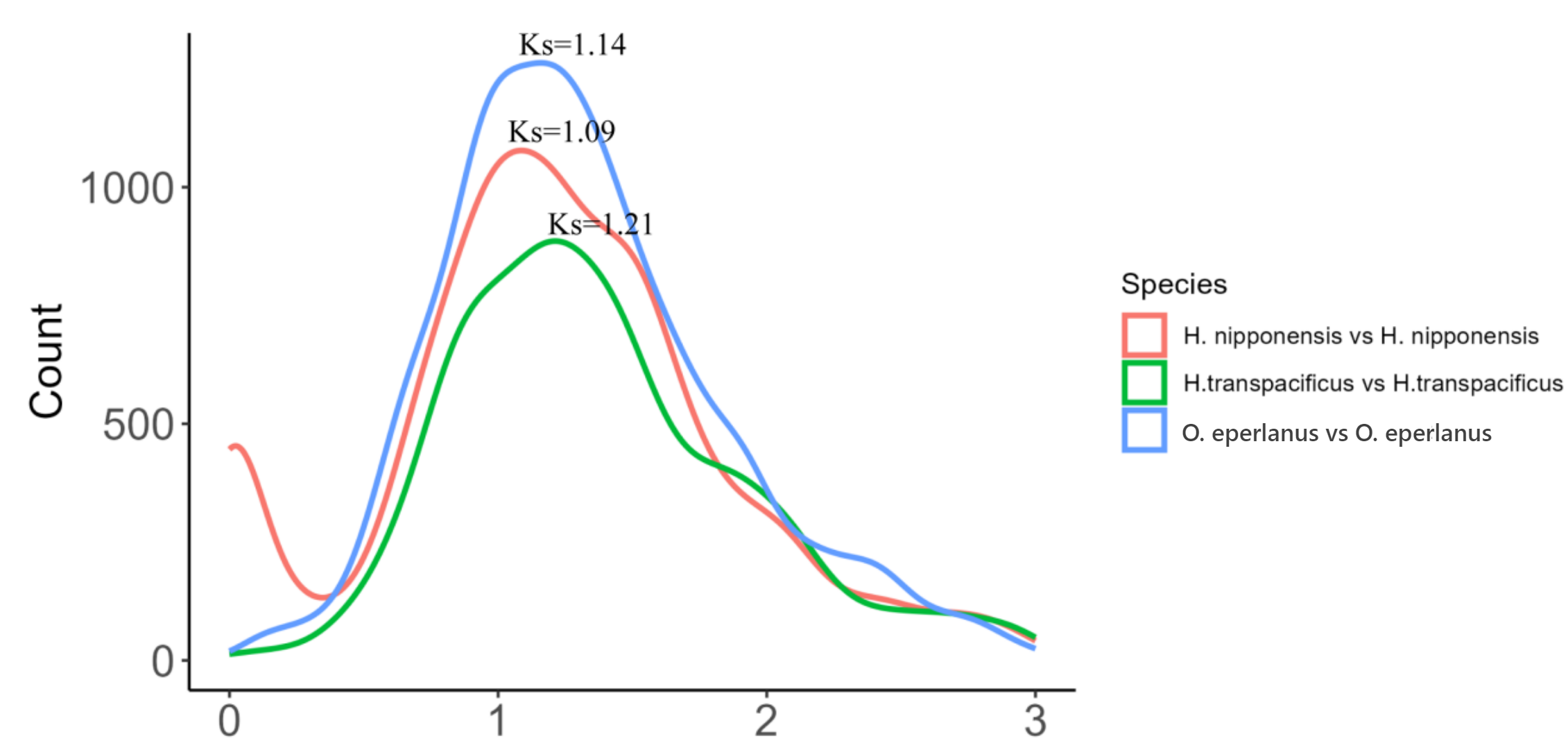
Supplementary Figure S5.** Density map of ks paralogous genes of *H. nipponensis,H. transpacificu and O. eperlanus*

**
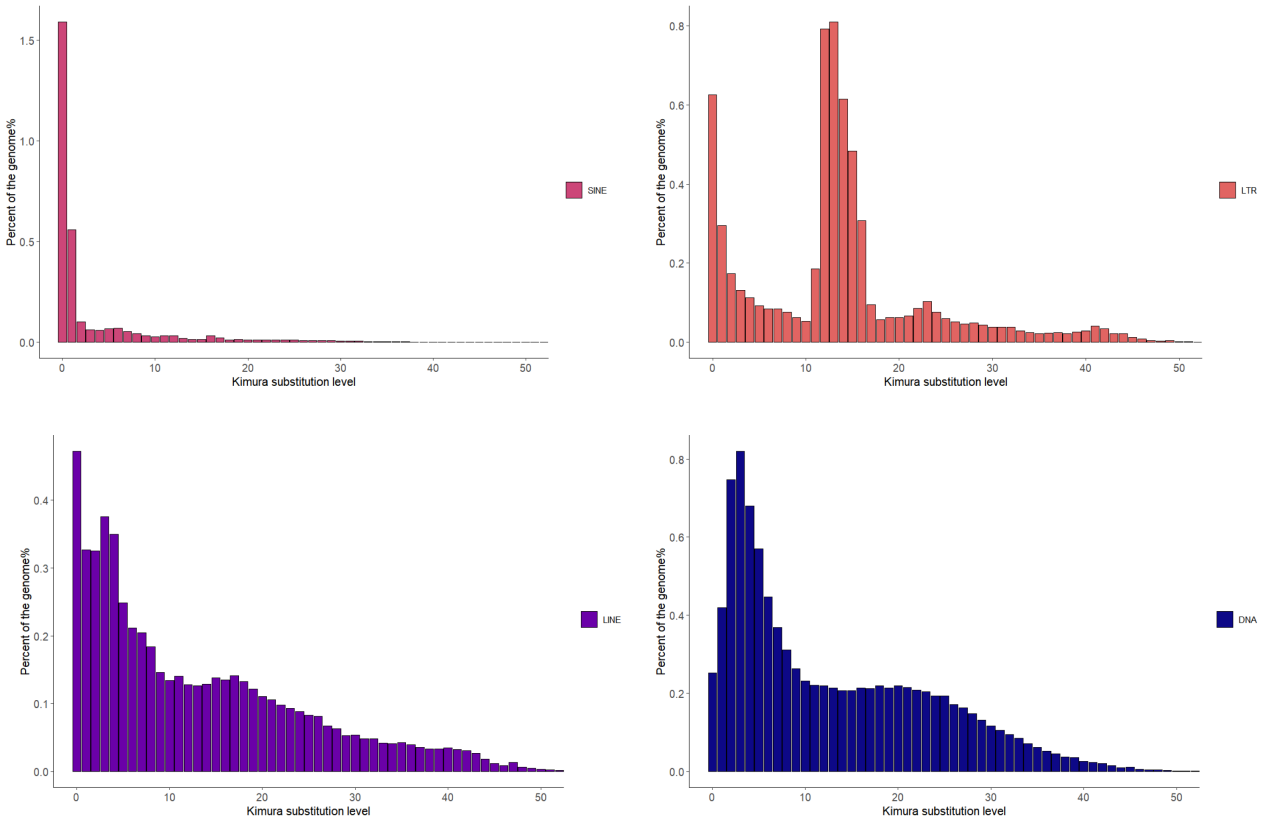
**

**Supplementary Figure S6.** The Kimura distance map of four transposons in *H. nipponensis*

**
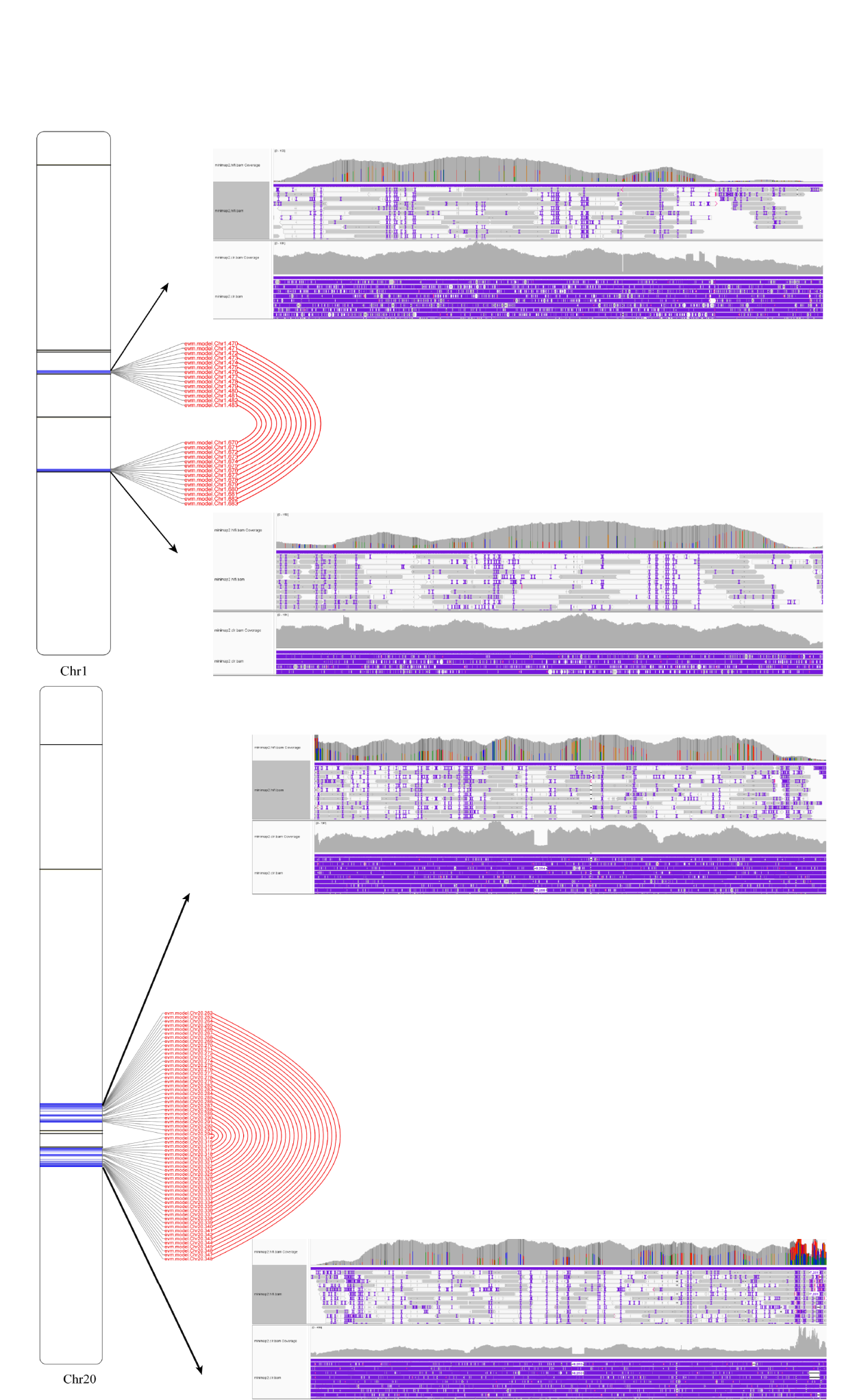
Supplementary Figure S7.** Schematic diagram of the recent amplified fragments of the paralogous gene family of *H. nipponensis*. evm.model. Chr1.470-483 and evm.model. Chr1 .670-683 are the corresponding amplified fragments. The evm.model.Chr1.262-294 and evm.model.Chr1.314-348 are the corresponding amplified fragments. The black line on the chromosome indicates N.


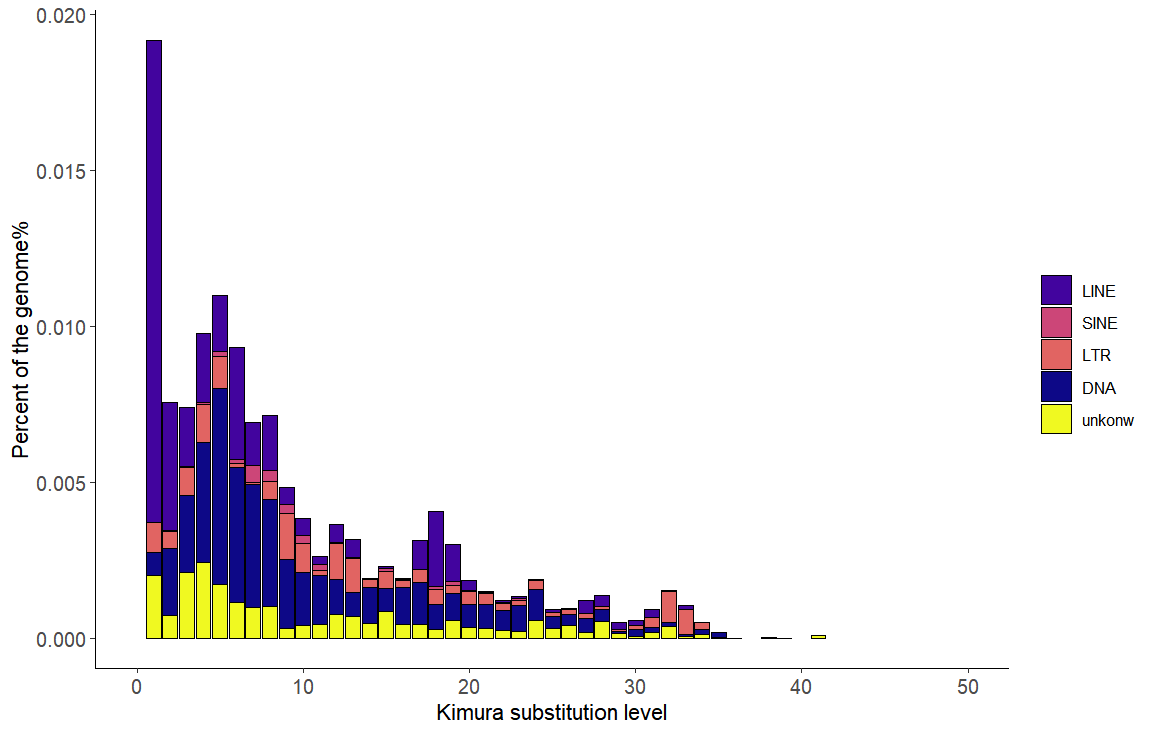
**Supplementary Figure S8.** Kimura distance map of repeated sequences within the expanded paralogous gene family of *H. nipponensis.*


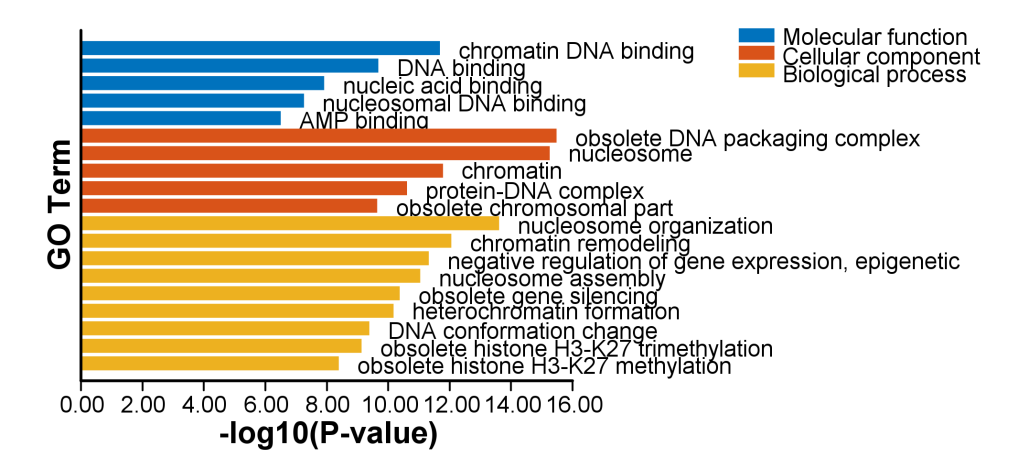


**Supplementary Figure S9.** *H. nipponensis* GO enrichment of recently expanded paralogous gene families


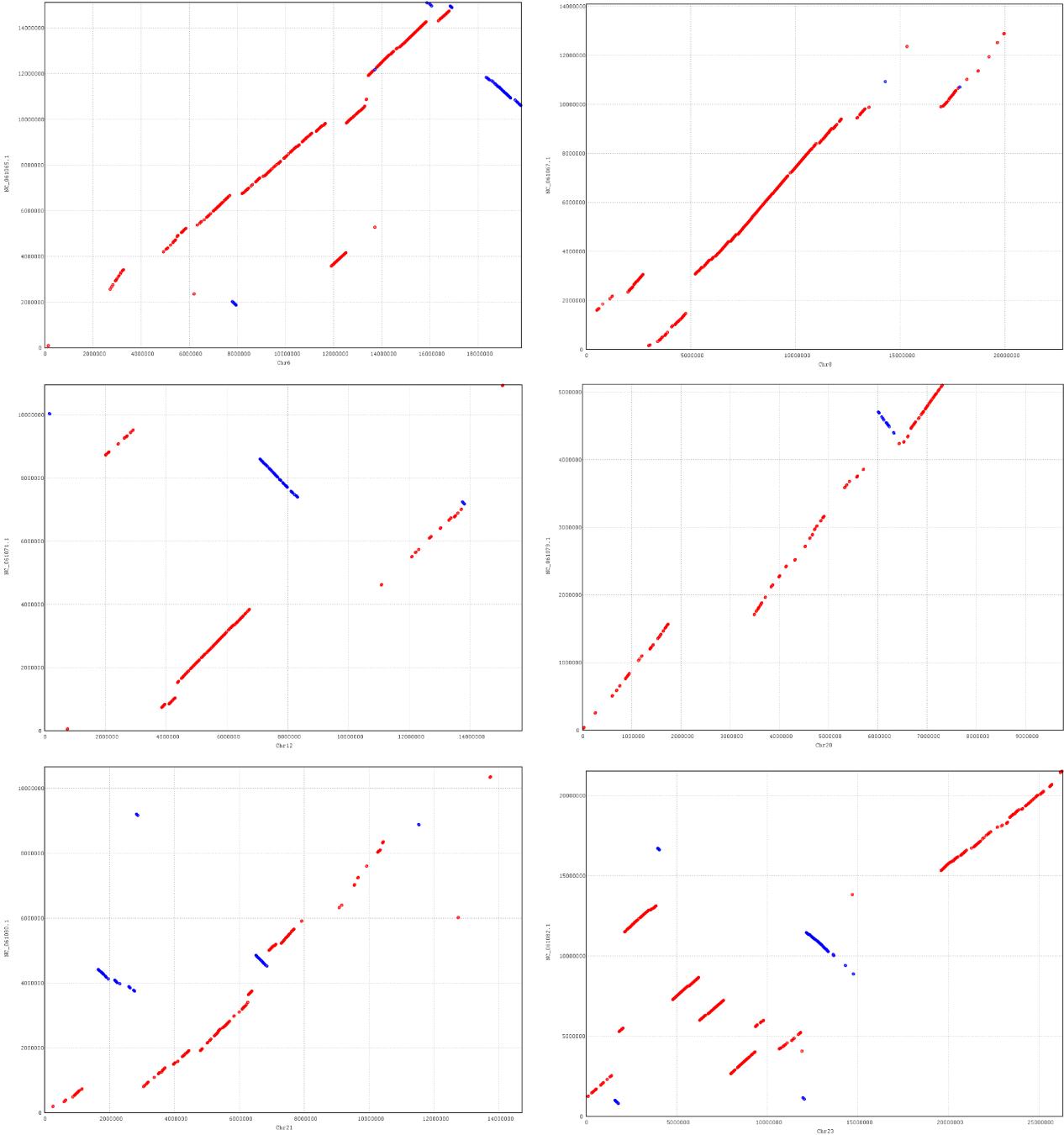


**Supplementary Figure S10.** Oxford lattice map of colinearity among the six chromosomes of *H. nipponensis* and *H. transpacificu*


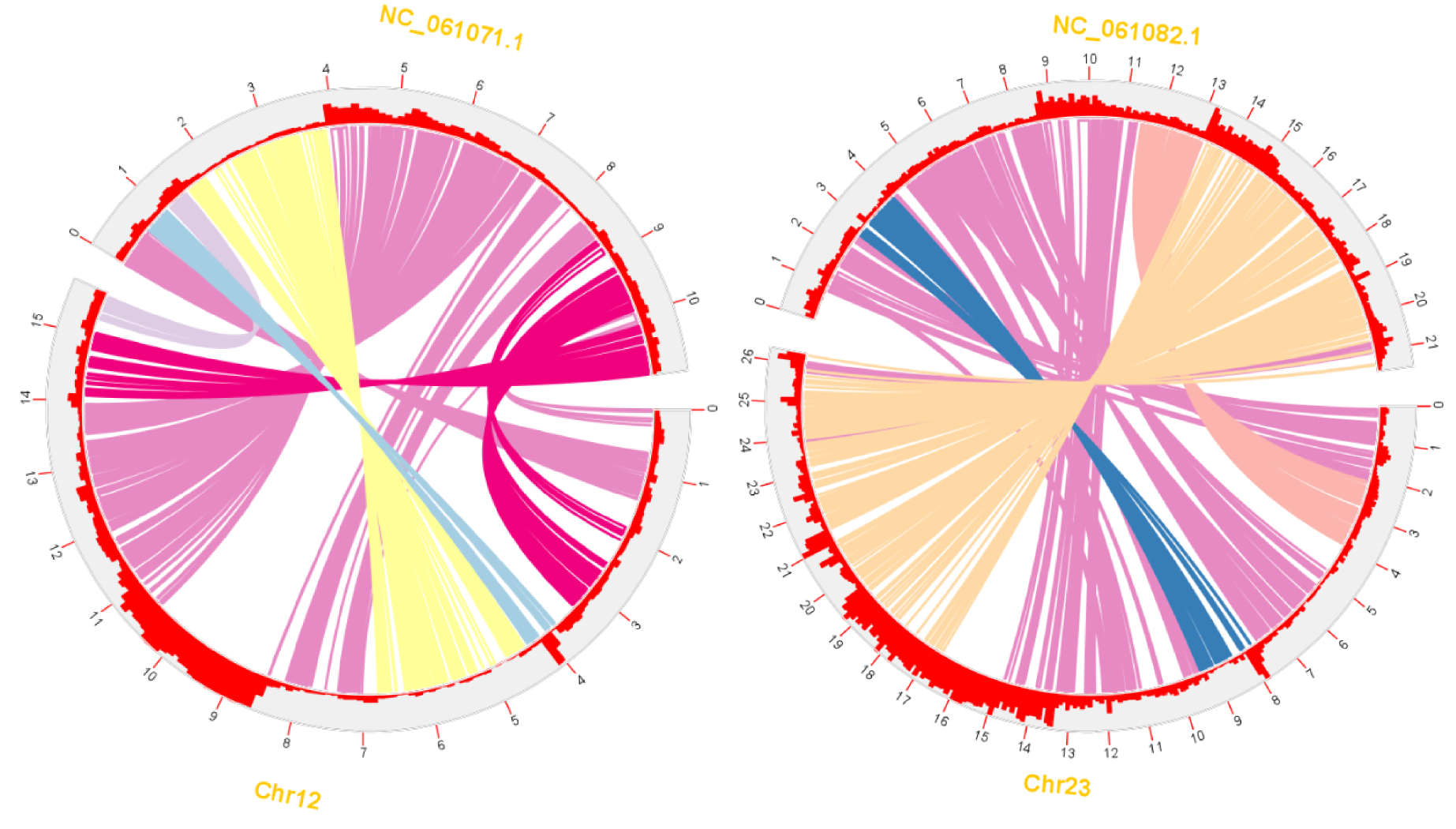
**Supplementary Figure S11.** Collinearity diagram of two chromosomes of *H. nipponensis* and *H. transpacificus*, with the outer circle representing the density of repeated sequences.

**
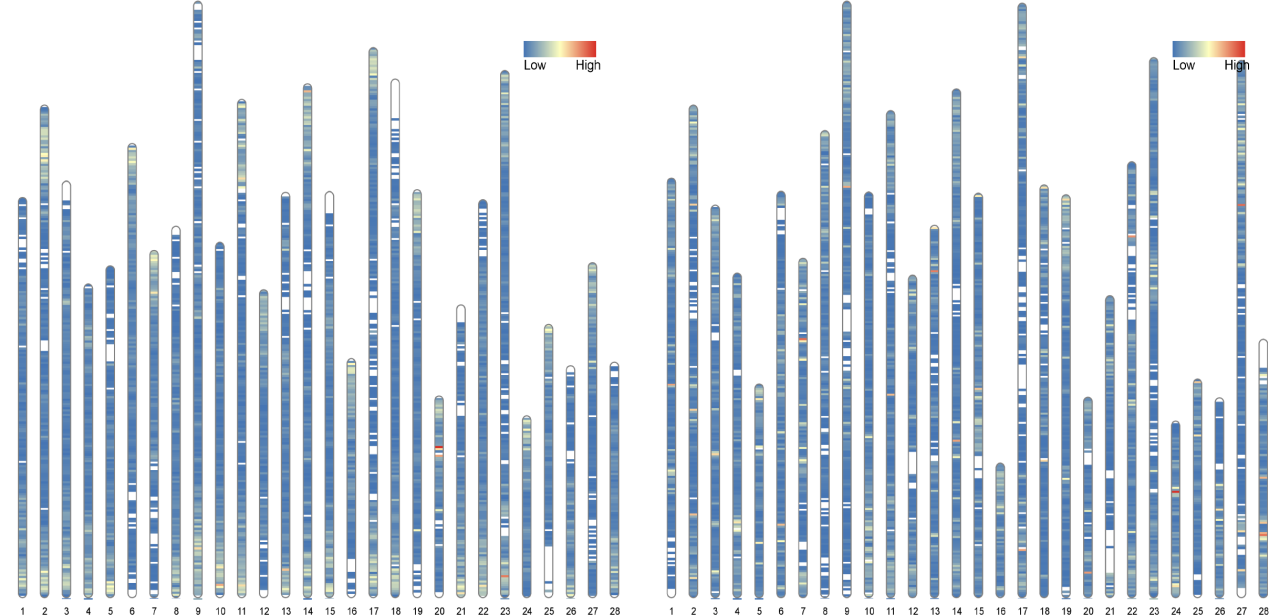
**

**Supplementary Figure S12.** The PAVs in *Hypomesus* and *O. eperlanus* genomes. For each chromosome, the left heatmap shows ratios of presence sequences in Hypomesus. The right heatmap shows ratios of presence sequences in *O. eperlanus*. Each heatmap is represented in 1 Mb windows sliding 200 kb

**
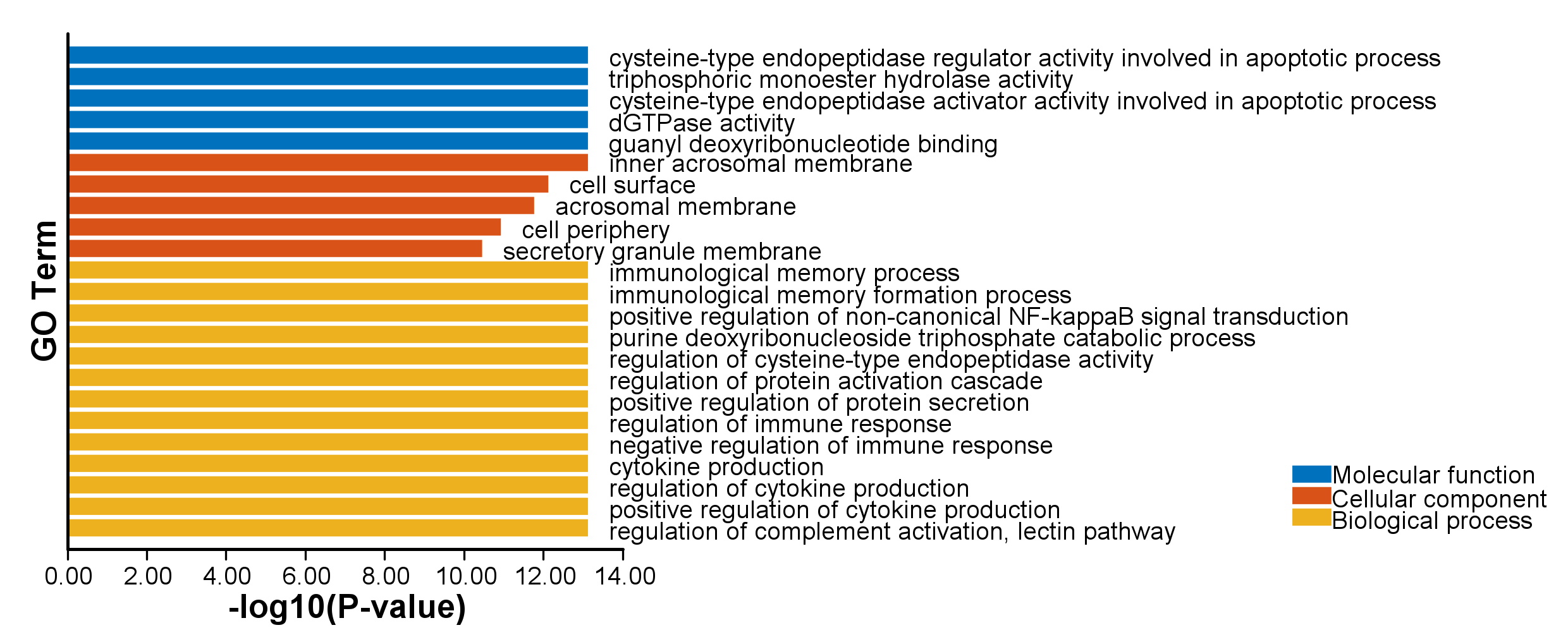
**

**Supplementary Figure S13.** *Osmerus eperlanus* Gene enrichment in the PAV region

Supplementary Table S1: Statistics of the *H. nipponensis* genome assembly

| Global statistics | Draft genome v0.1 | Draf genome v0.2 | Draft genome v0.3 | Draft genome v0.4 | Draft genome v0.5 | Genome sequence v0.6 |
| --- | --- | --- | --- | --- | --- | --- |
| **Genome assembly** |  |  |  |  |  |  |
| Number of contigs | 2508 | 561 | 392 | 376 | 375 | 375 |
| Total contig length(bp) | 501,179,557 | 494,832,257 | 530,745,426 | 531,337,140 | 532,586,180 | 532,586,180 |
| Contig length N50(bp) | 507,888 | 2,306,584 | 7,955,405 | 8,180,844 | 8,193,377 | 8,193,377 |
| Scaffold length N50(bp) | 507,888 | 2,306,584 | 7,955,405 | 8,180,844 | 8,193,377 | 20,113,295 |
| Longest contig (bp) | 7,749,587 | 12,060,874 | 20,367,250 | 21,267,153 | 21,290,931 | 21,290,931 |
| Average contig length(bp) | 199,832 | 3,033,868 | 1,353,942 | 1,413,130 | 1,420,229 | 1,420,229 |
| GC content(%) | 45.48 | 45.89 | 45.84 | 45.84 | 45.84 | 45.84 |
| N's per 100 k bp | 0 | 0 | 0.02 | 0.02 | 0.02 | 3.55 |
| **BUSCO statistics(%)** |  |  |  |  |  |  |
| BUSCO (Actinopterygii) complete | 92.8 | 95.6 | 95.7 | 96.3 | 96.8 | 96.7 |
| Complete and single-copy | 90.3 | 94 | 93.7 | 94 | 94.5 | 94.6 |
| Complete and duplicated | 2.5 | 1.6 | 2 | 2.3 | 2.3 | 2.1 |
| Fragmented | 1.7 | 1.6 | 1.1 | 1.2 | 1.1 | 1.1 |
| Missing | 5.5 | 2.8 | 3.2 | 2.5 | 2.1 | 2.2 |

| **Tissue samples** | **Experiment** | **Sample ID** | **Bases** | **Total number of reads** | **mapping rate %** |
| --- | --- | --- | --- | --- | --- |
| Liver | SRX9416072 | LF1 | 6,901,830,765 | 70,746,024 | 97.2 |
|  | SRX9416074 | LF3 | 8,650,458,186 | 86,110,502 | 97.88 |
|  | SRX9416075 | LF4 | 5,757,902,523 | 57,408,391 | 98.13 |
| Muscle | SRX9416066 | MF1 | 7,284,562,418 | 73,553,434 | 98.56 |
|  | SRX9416068 | MF3 | 6,112,854,742 | 61,956,377 | 98.26 |
|  | SRX9416069 | MF4 | 10,102,284,462 | 100,915,383 | 97.97 |

Supplementary **Table S2.** Basic characteristics of reads in 6 libraries and data for RNA-seq sequencing.

Supplementary **Table S3.** Published genome assemblies of the *Hypomesus*

| **Species** | **Reference** | **Size(Mbp)** | **Gene Number** | **Contig N50(Mbp)** | **Busco anotation assessment results** |
| --- | --- | --- | --- | --- | --- |
| ***Hypomesus  nipponensis*** | **This study** | **507M** | **27,876** | **8.0M** | **96.7%[S:94.5%,D:2.3%,],F:1.1%,M:2.1%,n:3640** |
| Hypomesus  transpacificus | PRJNA762373 (2022) | 437M | 27,592 | 0.40M | 92.2%[S:90.1%,D:2.1%,],F:0.7%,M:7.1%,n:3640 |
| *Hypomesus  nipponensis* | PRJNA672783 (2021) | 486M | 20,644 | 0.45M | 93.6%[S:85.3%,D:8.3%,],F:1.8%,M:4.5%,n:3640 |

**Supplementary Table S4**. Statistics of predicted gene structures.

| Gene Set | Number | Average Transcript Length (bp) |
| --- | --- | --- |
| Homolog |  |  |
| *Danio rerio* | 20,571 | 9,122.1 |
| *Gasterosteus aculeatus* | 13,165 | 8,347.4 |
| *Oryzias sinensis* | 16,230 | 9,797.7 |
| *Salmo salar* | 35,663 | 11,399.9 |
| *Takifugu rubripes* | 11,869 | 11,501.5 |
| *Hucho hucho* | 39,908 | 8,191.1 |
| Transcriptome | 22,485 | 10,110.7 |
| Breaker3 | 46,271 | 5,946.2 |
| EVidenceModeler | 27,876 | 8,122.5 |

**Supplementary Table S5.** The number of tandem repeats of different lengths in three species

| Specie | 100bp | 500bp | 1000bp | 2000bp | 5000bp | sum% |
| --- | --- | --- | --- | --- | --- | --- |
| *H.nippomensis* | 229694 | 32821 | 8899 | 2464 | 806 | 14.61 |
| *G.aculeatus* | 29205 | 6021 | 1602 | 398 | 100 | 3.26 |
| 1. *rubripes* | 53984 | 16369 | 5886 | 1381 | 190 | 5.38 |

**Supplementary Table S6.** NCBI accession numbers of fish genomes for the phylogenetic analysis.

| **Species** | **NCBI accession number** |
| --- | --- |
| *Danio rerio* | GCF_000002035.6 |
| *Oryzias latipes* | GCF_002234675.1 |
| *Esox lucius* | GCA_011004845.1 |
| *Callorhinchus milii* | GCA_018977255.1 |
| *Takifugu rubripes* | GCF_901000725.2 |
| *Protosalanx chinensis* | GCA_030340685.1 |
| *Neosalanx taihuensis* | GCA_030340665.1 |
| *Hypomesus transpacificus* | GCF_021917145.1 |
| *Osmerus eperlanus* | GCF_963692335.1 |
| *Salmo salar* | GCF_905237065.1 |
